# Gut Microbiome—Brain Crosstalk in the Early Life of Chicken Reveals the Circadian Regulation of Key Metabolic and Immune Signaling Processes

**DOI:** 10.3390/microorganisms13040789

**Published:** 2025-03-30

**Authors:** Mridula Gupta, Mustafa Cilkiz, Mohamed M. A. Ibrahim, Giridhar Athrey

**Affiliations:** 1 Department of Poultry Science, Texas A&M University, 2472 TAMU, College Station, TX 77843, USA; mridula28@tamu.edu; 2 Soil and Crop Sciences, Texas A&M University, College Station, TX 77843, USA; mcilkiz@tamu.edu; 3 Department of Laser Applications in Metrology, Photochemistry and Agriculture, National Institute of Laser Enhanced Sciences, Cairo University, Giza 12613, Egypt; mohamedmagdy@cu.edu.eg; 4 Faculty of Ecology & Evolutionary Biology, Texas A&M University, College Station, TX 77843, USA

**Keywords:** biological rhythms, chicken, RNA-sequencing, gut microbes, WGCNA, co-expression

## Abstract

Circadian rhythms are innate biological systems that control everyday behavior and physiology. Furthermore, bilateral interaction between the host’s circadian rhythm and the gut microbes influences a variety of health ramifications, including metabolic diseases, obesity, and mental health including GALT physiology and the microbiome population. Therefore, we are studying the correlation between differential gene expression in the chicken brain and microbiota abundance during circadian rhythms. To understand this, we raised freshly hatched chicks under two photoperiod treatments: normal photoperiod (NP = 12/12 LD) and extended photoperiod (EP 23/1 LD). The chicks were randomly assigned to one of two treatments. After 21 days of circadian entrainment, the chicks were euthanized at nine time points spaced six hours apart over 48 h to characterize the brain transcriptomes. Each sample’s RNA was extracted, and 36 mRNA libraries were generated and sequenced using Illumina technology, followed by data processing, count data generation, and differential gene expression analysis. We generated an average of 17.5 million reads per library for 237.9 M reads. When aligned to the Galgal6 reference genome, 11,867 genes had detectable expression levels, with a common dispersion value of 0.105. To identify the genes that follow 24 h rhythms, counts per million data were performed in DiscoRhythm. We discovered 577 genes with Cosinor and 417 with the JTK cycle algorithm that exhibit substantial rhythms. We used weighted gene co-expression network analysis (WGCNA) to analyze the correlation between differentially expressed genes and microbiota abundance. The most enriched pathways included aldosterone-regulated sodium reabsorption, endocrine and other factor-regulated calcium reabsorption, GABAergic synapse, oxidative phosphorylation, serotonergic synapse, dopaminergic synapse and circadian entrainment. This study builds on our previous study, and adds new findings about the specific interactions and co-regulation of the brain transcriptome and the gut microbiota. The interaction between gut microbiota and host gene expression highlights the potential benefits of microbiome-modulation approaches to improve gut health and performance in poultry.

## 1. Introduction

Circadian rhythms (from Latin “*circa*” = around and “*diem*” = day) are autonomous 24 h cycles that allow organisms to synchronize their internal biological rhythms with the external environment [1,2,3]. These oscillations driven by the circadian clock are observed in organisms, from single-celled to vertebrates [4]. These clocks internally regulate the numerous physiological processes of an organism via feedback loops controlled by transcription factors [5]. The circadian pacemakers in birds are the suprachiasmatic nucleus (SCN), pineal gland, and retina, in contrast to mammals, where the SCN is the sole pacemaker [6]. SCN is a master clock that constitutes about 20,000 neurons in the hypothalamus. It is driven by photoreception by the retinal cells. The molecular clock comprising *BMAL1/2*, *CLOCK*, *CRY1/2*, and *PER1/2/3* governs the transcription and rhythmic molecular and functional activity patterns [7]. Hence, disruption in circadian rhythms directly affects the body’s functioning at the cellular and molecular levels, which are implicated in various metabolic, immune, and neurodegenerative disorders [8,9,10,11]. In chickens, interruption in these oscillations directly impacts their physiological and reproductive health, therefore it is of utmost priority to optimize the light standards in the poultry industry. Chickens are ideal for studying circadian regulation of homeostasis and microbiota interactions due to their pentachromatic vision and uniqueness in detecting environmental rhythms. Their importance as a major livestock species and food source makes them a valuable target for continued improvement and optimization.

Circadian signaling impacts gut physiology through the vagus nerve and modulates immune cells in the Gut Associated Lymphoid Tissue (GALT) [12]. The circadian–gut interaction is a complex and bidirectional relationship maintaining homeostasis, including diurnal gut microbiome composition and function [13]. This synchronicity regulates metabolic responses to diet, lipid metabolism, and energy homeostasis [12,14,15,16,17]. Reciprocally, the gut microbiota influences the circadian clock, regulating the expression of clock genes and impacting the daily rhythmicity of the host [18,19]. One feature of gut dysbiosis is circadian asynchrony [20,21].

The bidirectional communication between the host circadian rhythm and the gut microbiota impacts various health outcomes, including metabolic disorders, obesity, and mental health [22]. The absence or disruption of gut microbiota profoundly affects brain function, behavior, and various molecular factors such as neuropeptide Y (NPY) and PYY [23]. Germ-free mice display neurochemical and functional changes compared to conventionally colonized mice. They also exhibit decreased expression of the NMDA receptor subunit 2A (NR2A) in the cortex and hippocampus [24] and reduced expression of the NR2B subunit and the 5-HT receptor 1A (5HT1A) in the central amygdala and the hippocampus [25]. In another study, a crucial neurotrophin, brain-derived neurotrophic factor (BDNF) expression was reduced in germ-free mice, highlighting the importance of a functioning microbiome for neuronal growth and survival [24,26]. These studies reflect the key interactions between neural factors, the gut microbiome, and GALT physiology. In avian species, there is emerging evidence of gut–brain crosstalk [27,28,29] suggesting mechanisms similar to those in mammalian models. Despite these studies, there are relatively few reports on the correlation between CLOCK-regulated expression and the gut microbiota, especially in early life, and recent studies have only begun to explore the implications of these interactions for metabolic and immune health in avian species.

Here, we show the crucial interactions and signaling pathways that are involved in the gut–brain crosstalk in the context of early-life circadian rhythms in chickens. We integrate brain transcriptome data with gut microbiota patterns from a circadian experiment to reveal the differing outcomes between functioning and dysfunctional circadian rhythms in early life. Furthermore, we relate co-expression and pathway activation data to highlight the bidirectional relationship between microbial abundance and key homeostatic signaling patterns.

## 2. Material and Methods

### 2.1. Animal Ethics Statement

The research was conducted in compliance with international and national standards for animal care. The Institutional Animal Care and Use Committee (IACUC) at Texas A&M University authorized and regulated the animal studies (Assurance Number 2016-0064).

### 2.2. Animals and Experimental Design

All the experimental details were previously reported in our paper Hieke et al. (2019) [30], but are briefly repeated here for clarity. Eighty hatch-day female chicks of the Hy-Line Brown Layer breed (*Gallus gallus domesticus*) were procured from a local hatchery (Bryan, TX, USA) and transported to the Texas A&M Poultry Research and Education Center in College Station, Texas. In each treatment, 40 chicks were randomly assigned to two experimental rooms with independent lighting controls. Within each room, 20 chicks were raised in one of two brooder cages. Each room was configured for a certain photoperiod treatment—normal photoperiod (NP) of 12 h of light and 12 h of darkness (12/12 LD), with lights-on at 06:00 h, and Extended treatment (EP) of 23 h L and 1 h D (23/1 LD), with lights-off from 05:00 to 06:00 h. Zeitgeber Time 0 (ZT0) was established as the time of lights-on (06:00 h), following convention. The experimental birds were raised in identical conditions except for photoperiod exposure, and provided with ad libitum access to food and water. A pullet diet containing 17% crude protein and 2800 kcal of metabolizable energy per kg was used to rear the chicks. The experimental rooms were temperature controlled, starting at 32 °C for a week and then gradually dropping by around 2–3 °C every week until reaching 23 °C, following standard chick-rearing protocols.

### 2.3. Sample Collection

Following circadian entrainment for 21 days, two birds were selected randomly (one from each brooder cage) at 6 h intervals for euthanasia and sampling. Chicks were euthanized by exposure to 5 min of CO_2_ followed by cervical dislocation. Two birds from each photoperiod treatment were sampled every 6 h (two individuals/treatment/time point) over a 48 h period, starting at ZT0 (a total of nine time points, a total of 18 per treatment). For sampling during the dark period (for NP treatment), the chicks were kept in a dark container during transport to another room for euthanasia. Brain samples were collected within 30 min of euthanasia and were placed into RNALater (Qiagen, Hilden, Germany) in a 1:5 ratio. As birds from both treatments had to be sampled at precisely the exact times, four individuals concurrently carried out the same procedures, from bird euthanasia to brain tissue collection within 30 min post-mortem. The sample tubes were kept at 4 °C for at least 24 h to ensure maximum absorption of RNALater. The RNAlater was removed after 24 h and samples were stored at −80 °C until RNA isolation. In our previous study, we used qPCR-based methods to assay the expression of specific circadian genes. Here, we generated the whole transcriptome dataset and analyzed its interactions with the microbiota data.

### 2.4. RNA Isolation and Quantification

From each individual, we collected 3 mm^3^ of brain tissue in the vicinity of the pineal gland, which was homogenized in Trizol reagent (Invitrogen, Carlsbad, CA, USA) using a Mini-Beadbeater-96 and 15–30 mg of 1.0 mm ZIRCONIA beads (cat. no. 11079124zx) (BioSpec, Bartlesville, OK, USA). We isolated total RNA and quantified RNA samples, and quality was assessed by evaluating protein contamination (260/280 ratio) and other organic contamination (230/260 ratio) using a NanoDropTM 1000 spectrophotometer Thermo Fisher Scientific, Waltham, MA, USA). The RNA integrity number (RIN) and suitability for library construction of the samples were evaluated using an Agilent RNA 6000 Nano kit (No:5067-1511) on a Bioanalyzer 2100 (Agilent Technologies, Inc., Santa Clara, CA, USA) chip reader. Using a QubitTM RNA BR assay (Thermo Fisher Scientific, Waltham, MA, USA), 20–1000 ng/L (Catalog number: Q10211), as well as a QubitTM dsDNA BR assay, 100 pg/L–1000 ng/L, we screened total RNA isolates with RIN 8.5 or above for genomic DNA contamination (Catalog number: Q32853). For library construction, total RNA samples that passed these quality criteria were normalized by diluting at 400 ng/L using nuclease-free water (NF water).

### 2.5. RNA Library Preparation and Transcriptome Profile Generation

We prepared the libraries from 200 ng of total RNA using the QuantSeq 3′ mRNA-Seq Library Prep Kit FWD for Illumina kit’s instructions (Lexogen, Vienna, Austria). We reverse-transcribed mature (poly-A-tailed) mRNA using oligo (dT) primers and Illumina-specific Read-2 linker sequences to create complementary first-strand DNA. Next, we synthesized the second strand of DNA using a random primer containing the Illumina-specific Read-1 linker sequence while the DNA polymerase enzyme was present. To eliminate contaminants that may hinder the library enrichment and indexing processes, we purified libraries utilizing a magnetic bead-based purification step. Cleaned libraries were normalized to 4 nM and valuated using the TapeStation 2200 equipment and the D1000 ScreenTape assay from Agilent Technologies, Inc, Santa Clara, CA, USA. At the Texas A&M Institute for Genome Sciences and Society (TIGSS, College Station, TX, USA), twenty-four libraries (N = 12/treatment) were pooled in equimolar concentrations and sequenced using an Illumina NextSeq (Illumina, San Diego, CA, USA) platform. Libraries yielded an average of 8.8 million reads in 75 bp single-end mode.

### 2.6. Transcriptome Data Analysis

We carried out all bioinformatics analysis with open-source tools using a well-established RNAseq analysis pipeline for read counts-based analyses. After removing adapter contamination and Lexogen indices, the single-end raw reads in FASTQ format were validated for quality with FastQC (Babraham Institute, Cambridge, UK) version 0.11.9 and MultiQC version 1.9 [31,32]. Adapter removal and quality filtering (Phred Q > 30), as well as the removal of reads under 35 bp, were performed using Trim_Galore version 0.4.5 [33]. Reads that passed quality filters were mapped to the *Gallus gallus* genome, Galgal6 (v6, Ensembl Release 99 GRCg6a, January 2020) [34] using the de novo splice mapper STAR (version STAR 2.5.3a modified). Using HTSeq-count (version 0.9.1) [35], we scored the single-end reads mapped to exon features. The obtained counts were normalized and analyzed to study differential gene expression in the package EdgeR using a two-factor model (version 3.26.8) on the R statistical platform (version 4.0.0) [36]. Low-expressed genes with expression < 1 CPM were excluded from the data for further analysis. To determine the oscillating genes, the CPM values were used to run DiscoRhythm analysis, a web-based application (https://mcarlucci.shinyapps.io/discorhythm/ (accessed on 6 July 2023) [37]. The genes from DiscoRhythm with *p*-value < 0.05 cut-off were pulled out from both algorithms, i.e., JTK and Cosinor. The genes from the Discorhythm were analyzed for the normal and extended photoperiods. These genes from both normal and extended photoperiods were analyzed using DiscoRhythm. Genes showing significant 24 h rhythms were further analyzed in ingenuity pathway analysis (IPA) [38,39], PathfindR [39], pathview Web [40] and cluster profiler [41] to determine which pathway genes are activated in the rhythms.

### 2.7. Microbiota Analysis

We used the OTUs obtained from the microbiota data from our previous study [30]. In this study, we extracted DNA fecal samples using the MoBio PowerFecal kit (Qiagen, Hilden, Germany) and the 16S rRNA gene was amplified with the bacteria using published primers [42] using Q5^®^ High-Fidelity DNA polymerase (NEBNext^®^ High-Fidelity 2X PCR Master Mix, New England BioLabs, Ipswich, MA, USA) with the necessary conditions [30]. On the Illumina MiSeq platform, the amplicons were sequenced and were processed in Mothur [43], aligning against the SILVA database [44]. Low-quality reads were filtered for quality, and chimeras were removed. Low-abundance OTUs were filtered at 0.01% and 1% thresholds. α and β diversity analyses were conducted in R using Phyloseq and vegan packages. Microbial differences between photoperiods were assessed via PERMANOVA, Metastats, and LEfSe.

### 2.8. Microbiota Abundance—Gene Co-Expression Quantification

To characterize the correlation between the differentially expressed genes and microbiome abundance, we used weighted gene co-expression network analysis (WGCNA) [45], to identify the genes and potential OTUs that are correlated under each photoperiod treatment. We further processed the data for dimensionality reduction using PCA and cluster analysis. The samples were clustered using the hierarchical cluster (hclust) function and Dynamic Tree Cut, and the network was constructed by selecting a soft thresholding of the correlation coefficient. We assessed the co-expression of circadian-regulated genes and microbiota abundance using blockwiseModules. The differentially expressed genes were assigned to different modules based on cluster analysis with the help of blockwiseModules and further analyzed their relationship with the individual OTUs.

## 3. Results and Discussion

### 3.1. The Differential Expression Analysis of Brain Tissue

**Figure 1:**
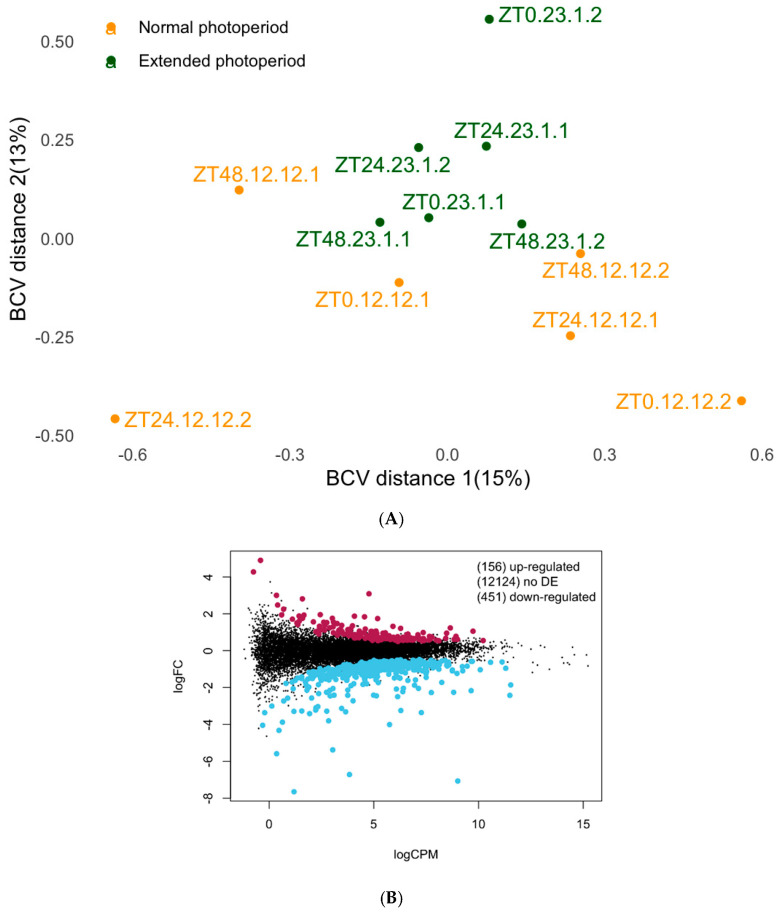
Edge R analysis: (**A**) Multidimensional scaling (MDS) plot of chicken brain transcriptomes raised under extended or normal photoperiod lighting regimens. Distances between the sample’s transcriptome profile correspond to the biological coefficient of variation (BCV) that represents the biological (nontechnical) variation. EP, Extended Photoperiod (23 h Light:1 h Dark), and NP, Normal Photoperiod (12 h light:12 h Dark). (**B**) A mean difference (MD) plot displaying the log2fold change (*y*-axis) versus average abundance (logCPM, *x*-axis) for each gene, comparing the differences between the extended and normal photoperiod in newly hatched chicks. Significantly up or down DE genes (FDR < 0.05) are highlighted in maroon and sky blue, respectively.

**Figure 2:**
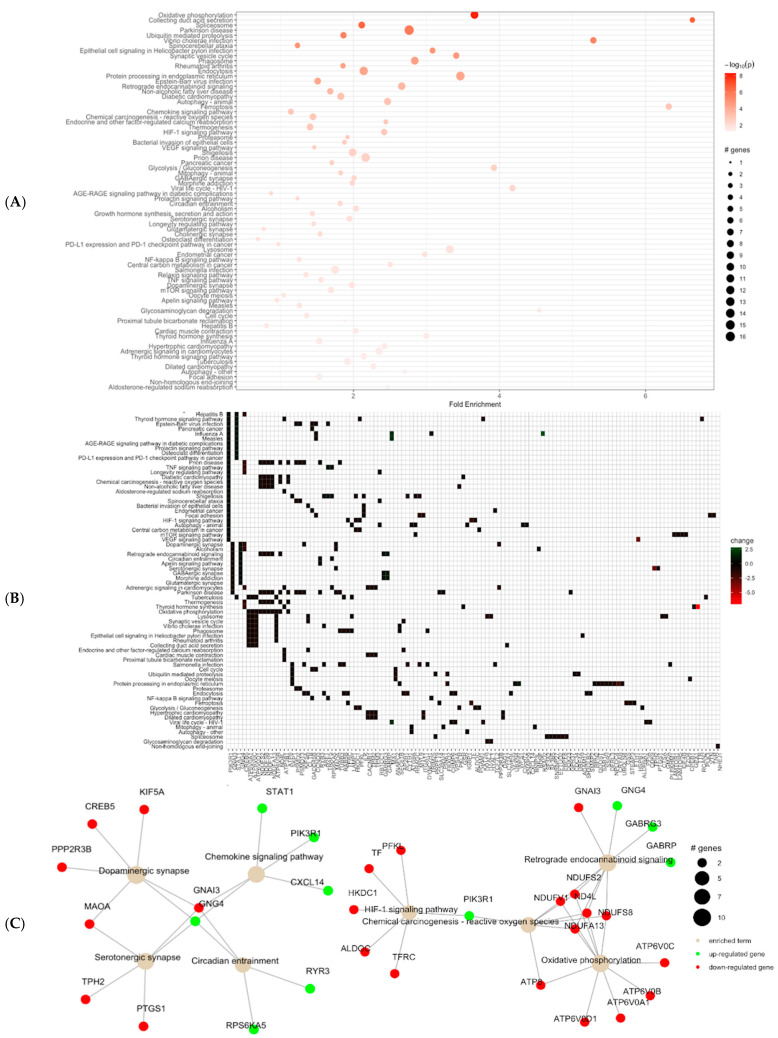
Pathway enrichment analysis using pathfinder. (**A**) Pathway enrichment analysis of RNAseq brain data for the normal and extended photoperiods of chickens in pathfindR. (**B**) Visualization of the pathway terms by the Gene IDs. (**C**) Visualization of selected enriched pathway terms and gene interactions with each other (green nodes represent upregulated genes, and red nodes represent downregulated genes).

**Figure 3:**
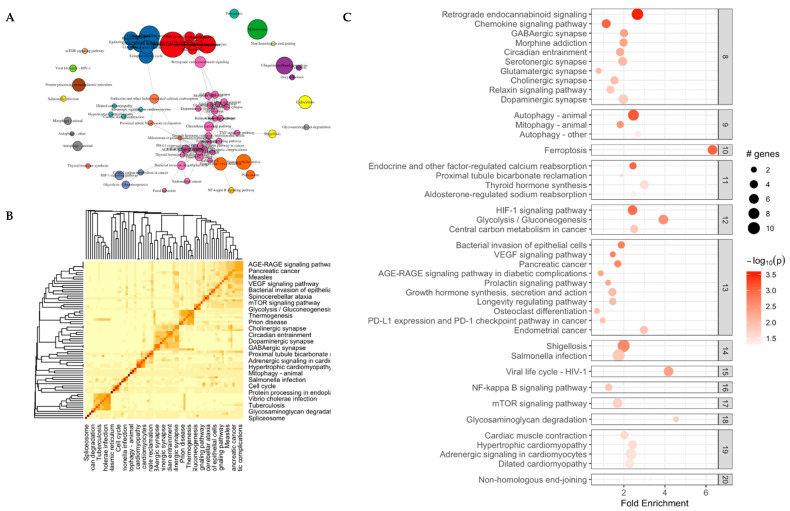
Clustering results for the output of pathway enrichment by PathfindR. (**A**) Visualization of pathway clusters in clustering graphs for normal and extended photoperiods. The size of each node corresponds to its −log(lowest-*p*) in an enriched pathway. (**B**) The heatmap represents the kappa statistic matrix graph for the chance-corrected measure of co-occurrence between normal and extended photoperiods. (**C**) Bubble chart displaying enrichment results, with clusters labeled on the right side of each panel. The *x*-axis represents fold enrichment values, while the *y*-axis indicates enriched pathways. Bubble size corresponds to the number of differentially expressed genes (DEGs) in each pathway. The color indicates the −log10(lowest-*p*) value, with darker red shades indicating higher significance of pathway enrichment.

To analyze the role of photoperiods and circadian rhythms on gene expression patterns in the brain, we focused on samples collected at ZT0 (06:00 h local time) (Figure 1A). We analyzed 12 RNAseq libraries from brain tissue, with six samples from each photoperiod treatment (N = 6). We generated an average of 17.5M reads per library, totaling 237933593.3 reads (Table 1). When aligned against the Galgal6 reference genome [Ensembl 99 release version], 11,867 genes showed detectable expression levels, and the dataset showed a common dispersion estimate of 0.105. Of the expressed genes, 607 genes were differentially expressed between the normal and extended photoperiod treatments at the ZT0 timepoint (6 a.m.). Among these differentially expressed genes (DEGs), 451 genes were downregulated, and 156 genes were upregulated in the normal photoperiods (Figure 1B). Ingenuity Pathway Analysis (Qiagen, Hilden, Germany) showed that Dopamine degradation, S100g family signaling pathway, Mitochondrial dysfunction, G-protein coupled receptor signaling, and Serotonin degradation were downregulated in extended photoperiods. PathfindR results showed the most activated pathways were Aldosterone-regulated sodium reabsorption, Endocrine and other factor-regulated calcium reabsorption, GABAergic synapse, Oxidative phosphorylation, Serotonergic synapse, Dopaminergic synapse, Circadian entrainment, and Ferroptosis. with the Fold enrichment ranging between 1.5 and 6.6 (Figure 2). The cluster enrichment analysis performed with the hierarchical clustering method grouped the pathways based on molecular function (Figure 3).

The co-activation of various pathways with circadian pathways is not surprising, given the central role of the circadian in homeostasis [46]. For instance, the enzyme monoamine oxidase A (MAOa) plays a crucial role in dopamine metabolism and is under clock control (Figure 2) and impacts mood-related behaviors such as depression and addiction [47]. In addition to synthesis and metabolism, dopamine release contributes significantly to circadian rhythms in behavior and physiology. Melatonin release from the pineal gland exhibits robust rhythmicity and is commonly used as a circadian marker. Melatonin release depends on the heteromerization of adrenergic receptors with dopamine D4 receptors, underscoring the pivotal role of dopamine in regulating pineal function [48]. Dopamine neuroendocrine neurons also express circadian clock genes, contributing to the circadian regulation in the host organism. The activation of the dopamine metabolism and circadian pathways in the NP treatment suggests the tight coordination of these systems—a sign of a functioning circadian [49].

Mitochondrial functioning regulated under circadian activity is a dynamic process crucial for maintaining cellular homeostasis and overall health [50]. Mitochondria plays a role in regulating cellular redox balance, and circadian disruption impacts mitochondrial respiration, which may induce hypoxia regulated by the HIF-1 signaling pathway [51]. In Figure 3, the HIF-1 signaling pathway is co-expressed with the oxidative phosphorylation and retrograde endocannabinoid signaling which implies that the perturbations in circadian rhythms can affect mitochondrial integrity and function, exacerbating oxidative stress and impairing cellular bioenergetics, driving a surge of reactive oxygen species (ROS) [52]. These interactions underscore the interconnectedness of circadian-regulated processes, including mitochondrial function and ROS signaling in orchestrating cellular physiology. We also found the activation of the ferroptosis pathway, which is a term associated with programmed cell death characterized by iron-dependent lipid peroxidation [53]. Circadian rhythms regulate ferroptosis through clock proteins like ARNTL/BMAL1, which suppress lipid peroxidation and oxidative stress by activating antioxidant pathways. Diurnal variations in iron metabolism, antioxidant systems, and cellular stress responses can modulate ferroptotic susceptibility [54]. A functioning circadian clock may upregulate ferroptosis during specific phases, promoting the clearance of damaged cells and aligning cellular turnover with environmental cycles for optimal tissue homeostasis. Our observation of this pathway also illuminates the role of the circadian in regulating autophagy at periodic intervals, which can be a key part of maintaining homeostasis and stress adaptation [55]

### 3.2. Genes Following Circadian Rhythm

**Figure 4:**
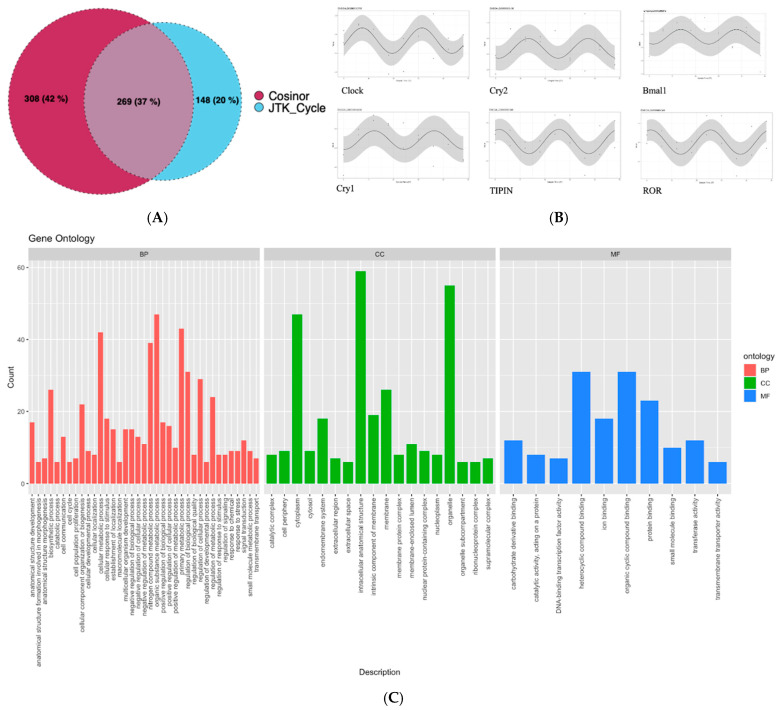
DiscoRhythm analysis: (**A**) The number of genes showing significant (*p* < 0.05) oscillating patterns with 24 h rhythms in the normal photoperiod treatments in chicken brain cells determined by Cosinor and JTK_Cycle algorithms. 269 genes were found to be oscillating in both methods, shown in the purple shaded area. (**B**) The oscillating patterns of core circadian oscillators showing significant rhythmic expression activity, showing the (**C**) Gene Ontology terms for the genes involved in circadian rhythm and showing rhythmic activity.

We analyzed expression intensities (CPM) of the samples from NP and EP treatments in DiscoRhythm to identify genes showing diurnal oscillations (Figure 4B). We found 577 oscillating genes with Cosinor and 417 with the JTK-cycle algorithm showing significant 24 h rhythms (*p* < 0.05) (Figure 4A). These two sets accounted for 723 unique genes, of which 269 were shared between the two algorithms (Supplementary Files S1 and S2).

We assessed the 723 oscillating genes for gene ontology germs. “The organic substance metabolic process”, “primary metabolic process”, and “cellular metabolic process” were the top three enriched biological processes with the highest gene counts involved. The top three enriched cellular components with the highest gene counts included “intracellular anatomical structure”, “organelle”, and “cytoplasm”. The top enriched molecular functions were “heterocyclic compound binding”, “organic cyclic compound binding”, and “protein binding” (Figure 4C).

Furthermore, to investigate differences between the NP and EP treatments, we subset the data for ZT0 which corresponds to the start of the light phase. These genes were enriched for canonical pathways FAK signaling, Phagosome formation, and Caveolar-mediated endocytosis signaling (activation Z-score > 2). The primary clock genes such as *CLOCK*, *CRY1/2*, *BMAL1*, *ROR,* and *TIPIN* and four GPCR receptors F*GFR*, *SUCNR1*, *HTR5*, *CXCL14*, *TACR3* showed robust 24 h rhythms. Additionally, these receptor genes mediate G-protein coupled receptor signaling, which includes tachykinin signaling, hydroxytryptamine receptor signaling, cytokine signaling, and phagosome formation, which also showed 24 h rhythms. These genes have a role in the overall immune health of the host [56,57,58,59] normal immune signaling. The activation of SUCNR1 suggests the invocation of succinate, a versatile compound that functions like hormones and cytokines [60]. *SUCNR1* regulates metabolism by modulating circadian signaling and leptin expression, and *SUCNR1*-deficient adipocytes disrupt the body’s ability to respond to leptin after feeding [61]. Succinate, a significant component within the tricarboxylic acid cycle, also triggers the production of inflammatory cytokines such as *IL-1β*, *IL-6*, *IL-8*, and *TNF-α* [62]. Secondly, *HTR* signaling is responsible for serotonin release, and its expression is clock- dependent [63]. Serotonin also immunomodulates immune cells by secreting cytokines. Serotonin suppresses pro-inflammatory cytokines such as *TNF-A* and *IL-1b* [64,65]. Another chemokine under clock control is CXCL14, which is secreted by a broad range of cells (immune and non-immune) and modulates inflammatory responses through GPCRs [58]. Our findings of these interactions in the early life of a chick, and during the window of circadian entrainment and microbiome assembly, imply a close coordination of microbiota-mediated processes with circadian-regulated genes.

### 3.3. Correlation of Gene Expression and Gut Microbiota

**Figure 5:**
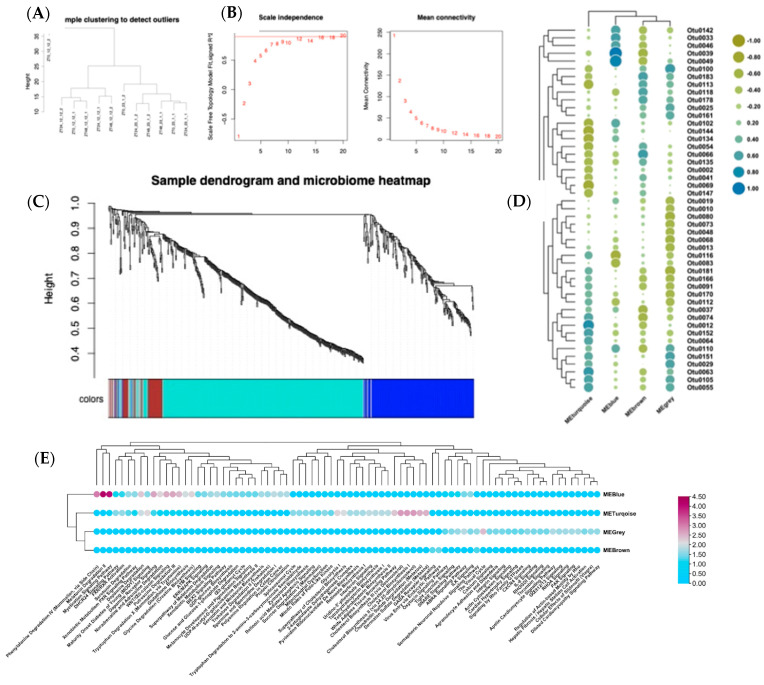
WGCNA analysis: (**A**) A dendrogram showing the clustering of the normal and extended photoperiods using hierarchical clustering (**B**) Network topology analysis to pick a soft threshold value. (**C**) The genes were assigned into clusters using blockwise modules. (**D**) Correlation between modules. The modules METurquoise, MEBlue, MEbrwon, MEgrey and different Operational Taxonomical Unit (OTUs are shown. (**E**) Top enriched pathways in each module based on the *p*-value (IPA output).

**Figure 6:**
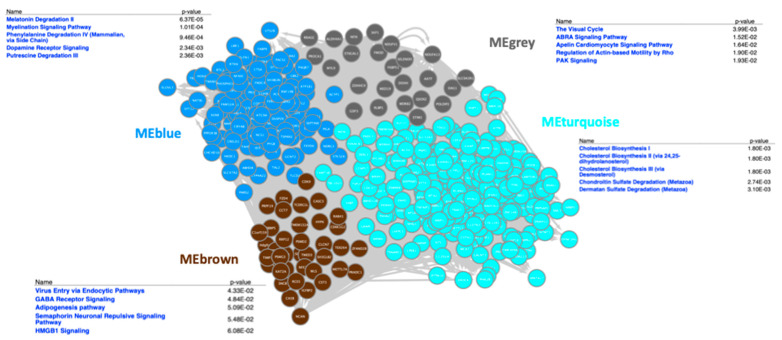
Gene-regulatory network in GeneMania against human genome, and top canonical pathways from IPA in each module.

To investigate the specific interactions between circadian-regulated gene expression and the microbiota, we performed co-expression analysis using the tool WGCNA integrating the RNAseq data generated here and microbiota data from our previous study [30]. The hierarchical clustering (using ‘hclust’) showed the normal and photoperiod treatments formed two distinct clusters (Figure 5A). The pickSoftThreshold was used for network topology analysis and was set as power 5 (Figure 5B). Based on this power, the gene expression modules for each sample were clustered into four modules such as MEbrown, MEturquoise, MEblue, and MEgrey (Figure 5C and Figure 6). Each module defines the correlation between each OTU and signifies the module–microbiome relationship (Figure 5D) (Supplementary Table S3).

The relative abundance of specific OTUs was correlated with the turquoise module, enriched for cholesterol biosynthesis and mitochondrial dysfunction. The relative abundance of *OTU069* (*Lactobacillus_*72.9%. *p* = 0.007), OTU144 (*Lachnospiraceae_NC2004*, 71.9%, *p* = 0.008), Otu0134 (*Defluviitaleaceae_UCG-011*, 67.3%, *p* = 0.016), Otu0102 (*Lachnospiraceae_FCS020_group*, 62.1%, *p* = 0.03), Otu0113 (*Ruminococcaceae_UCG-002*, 61.9%, *p* = 0.03), Otu0054 (*Clostridiales_vadinBB60_group*, 60.4%, *p* = 0.037) was negatively correlated with the turquoise module. This means that these bacterial groups may play protective or inhibitory roles against pathways related to cholesterol metabolism or mitochondrial dysfunction. On the other hand, MEturquoise is positively correlated with relative abundance of Otu0055 (*Gastranaerophilales_unclassified*, 58.6%, *p* = 0.04), OTU0012 (*Alistipes*, 76.4%, *p* = 0.003), Otu0105 (*Ruminococcaceae_UCG-014*, 58.6%, *p* = 0.04), Otu0152 (*Ruminiclostridium_9*, 60.8%, *p* = 0.03), Otu0063 (*Ruminococcaceae_UCG-014*, 68.1%, *p* = 0.014). This positive correlation suggests these microbes might actively promote or contribute to metabolic changes leading to elevated cholesterol synthesis and mitochondrial impairment.

The correlations between the expressed genes and taxa in the turquoise module highlight the coordination between microbiota members and homeostatic functions such as cholesterol biosynthesis and mitochondrial function. The implications of these correlations can be understood through the importance of cholesterol biogenesis for normal function. Altered cholesterol is associated with mitochondrial dysfunction and subsequent ATP deficiency, impacting neuronal function [66]. Additionally, mitochondrial fusion and ERK activity are crucial in cholesterol transport, affecting steroidogenesis [67].

Mitochondrial dysfunction is also sensitive to cholesterol levels, as studies have shown that external cholesterol levels impact mitochondrial function and inflammatory responses [68]. Furthermore, mitochondrial membrane transporter deficiencies lead to iron imbalance, affecting cholesterol biosynthesis and metabolic pathways [69]. The circadian regulation of this crucial bidirectional interaction between cholesterol metabolism and mitochondrial function points to the important interactions indicated by this module. The correlation of these functions with specific microbial taxa in the turquoise module further suggests the circadian-regulated microbiota–homeostatic signaling crosstalk.

In our previous studies [30], Rikenellaceae (Alistipes), Lachnospiraceae, and Ruminococcaceae were dominant in EP. Alistipes contributes to the production of saturated fatty acids [70], aligning with the turquoise module’s most enriched pathways: cholesterol biosynthesis and mitochondrial dysfunction [71]. In the NP treatment group, Lactobacillus abundance correlated with the expression of cholesterol-regulating genes NPC1L1, CYP7A1, and ABCG5, previously characterized in humans. This correlation suggests Lactobacillus abundance may influence cholesterol biosynthesis and mitochondrial dysfunction during microbiome assembly in chickens. The differential enrichment and correlation of Lactobacillus with cholesterol biosynthesis pathways could be explained by probiotic effects on lipid metabolism. Certain Lactobacillus strains influence cholesterol assimilation and bile acid concentrations [72,73], with evidence showing they can reduce serum total bile acid levels, thereby impacting cholesterol metabolism [74]. Specific strains, such as Lactobacillus fermentum and Lactobacillus plantarum, have demonstrated probiotic effects on hypercholesterolemia, effectively lowering cholesterol levels in animal models [75,76]. These findings support a potential mechanistic role of Lactobacillus strains in modulating cholesterol metabolism both in vitro and in vivo [76].

The blue module was negatively correlated with Otu0116 (*Ruminococcaceae_UCG-004*, 7.5%, *p* = 0.016), and positively correlated with Otu0046 (*Ruminococcaceae_UCG-014*, 58.1%, *p* = 0.047), OTU0142 (*Lachnospiraceae*, 59.01%, *p* = 0.043), Otu0033 (*Flavonifractor*, 63.5%, *p* = 0.03), OTU0039 (*Clostridiales vadinBB60*, 86%, *p* = 5.68 × 10^−9^), and OTU0 049 (*Eubacterium coprostanoligenes*, 98%, *p* = 0.0002).

The most enriched canonical pathways in blue modules were dopamine receptor signaling, melatonin degradation, myelination signaling pathway, and WNT/β-catenin signaling pathway analyzed in IPA (Figure 5E).

The enrichment of canonical pathways such as dopamine receptor signaling, melatonin degradation, myelination signaling pathway, and WNT/β-catenin signaling pathway in the blue module points to the functional roles of microbiota members (Figure 5E). These pathways play crucial roles in neurotransmission, circadian rhythm regulation, myelin formation, and cell signaling. The negative correlation with Otu0116 (*Ruminococcaceae*_UCG-004) may suggest a potential regulatory role of this taxon in modulating dopamine receptor signaling, melatonin degradation, myelination signaling pathway, and WNT/β-catenin signaling pathways. The presence of *Ruminococcaceae* and *Lachnospiraceae* taxa in the positive correlations is noteworthy, as these families are known for their roles in promoting gut health and metabolism. Flavonifractor, Clostridiales vadinBB60, and *Eubacterium coprostanoligenes* are also important gut microbiota members with potential implications for host physiology and metabolism. In chickens, there is also evidence for the influence of these taxa on behavior: cecal microbiota transplantation with *Lachnospiraceae* and *Ruminococcaceae UCG-005* in chickens at an early promoted aggressive behavior in recipient chickens by controlling the functions of the catecholaminergic and serotonergic systems in the brain [77].

The grey module was negatively correlated with Otu0181 (*Ruminococcaceae*, 66.%2, *p* = 0.0188), Otu0080 (*Oscillibacter*, 64.8%, *p* = 0.022), and Otu0068 (*Lachnospiraceae*, 60.9%, *p* = 0.03). The grey module was positively correlated with Otu0029 (*Ruminococcaceae_UCG-014*, 58.2%, *p* = 0.04), Otu0151 (*Christensenellaceae_R-7_group*, 60.4%, *p* = 0.03), and Otu0100 (*Lachnospiraceae*, 66.2%, *p* = 0.01) and was significant. The enriched pathways in this module are the Visual Cycle, ABRA signaling pathway, Apelin cardiomyocyte signaling pathway, and regulation of actin-based motility by Rho (Figure 5E).

The negative correlations with Otu0181 (*Ruminococcaceae*), Otu0080 (*Oscillibacter*), and Otu0068 (*Lachnospiraceae*) suggest a potential regulatory role of these taxa in modulating the pathways associated with the Visual Cycle, ABRA signaling pathway, Apelin cardiomyocyte signaling pathway, and regulation of actin-based motility by Rho within the grey module. On the other hand, the positive correlations with Otu0029 (*Ruminococcaceae_UCG-01*4), Otu0151 (*Christensenellaceae_R*-7_group), and Otu0100 (*Lachnospiraceae*) indicate a potential contribution of these microbial taxa to the activation or enhancement of these pathways. The Apelin–APJ pathway can directly antagonize vascular disease-related Ang II actions [78], providing insights into the regulatory mechanisms of the apelin signaling pathway in cardiovascular health. The microbiota associations suggest intriguing hypotheses about how early-life microbiome interactions with host homeostatic pathways can affect multiple major functions.

The brown module is negatively correlated significantly (*p* < 0.05) with Otu0039 (*Clostridiales vadinBB60*_group, 60.3%, *p* = 0.03), Otu0037 (*Clostridiales_vadinBB60_group*, 58.9%, *p* = 0.04), Otu0074 (*Ruminococcaceae_UCG-014*, 8.07%, *p* = 0.04, and positively correlated with Otu0118 (*Anaerotruncus*, 57.7%, *p* = 0.03), Otu0183 (*Ruminiclostridium_9*, 61.26%, *p* = 0.03), and Otu0066 (*[Eubacterium]_hallii_group*, 64.8%, *p* = 0.022). The top canonical pathways in this module are the Virus Entry via Endocytic Pathways, GABA Receptor Signaling, Adipogenesis pathway, Semaphorin Neuronal Repulsive Signaling Pathway, HMGB1 Signaling (Figure 5E).

The interactions observed within the brown module, as indicated by the significant correlations with specific OTUs, suggest a complex interplay. The negative correlations with OTUs such as Otu0039 (Clostridiales vadinBB60_group), Otu0037 (Clostridiales_vadinBB60_group), and Otu0074 (Ruminococcaceae_UCG-014) may indicate a potential regulatory role of these taxa in modulating pathways associated with the Virus Entry via Endocytic Pathways, GABA Receptor Signaling, Adipogenesis pathway, Semaphorin Neuronal Repulsive Signaling Pathway, and HMGB1 Signaling within the brown module. Altogether, these modules indicate intriguing correlations between the microbiota and transcriptome in the context of circadian rhythms, and point to the foundational role these interactions may have on later life metabolic and immune health.

### 3.4. KEGG Pathway Analysis

**Figure 7:**
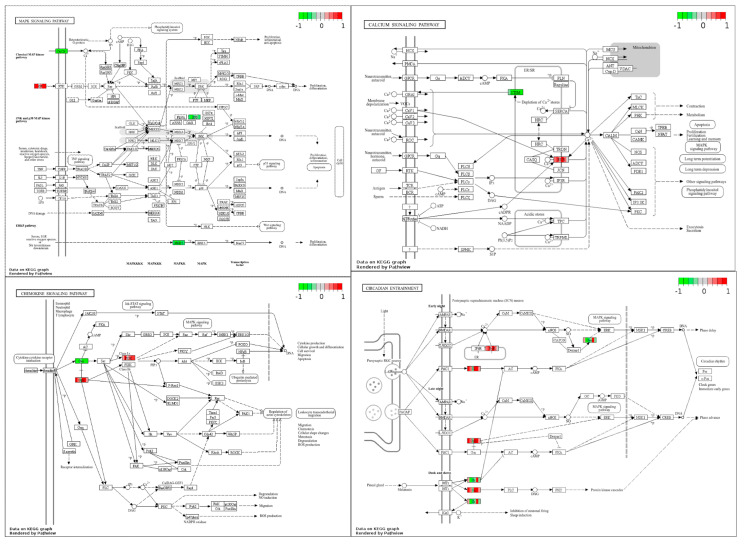
The genes queried against the KEGG database using Pathview, which show the activated genes in the MAPK, calcium signaling, chemokine signaling, and circadian entrainment pathways.

**Figure 8:**
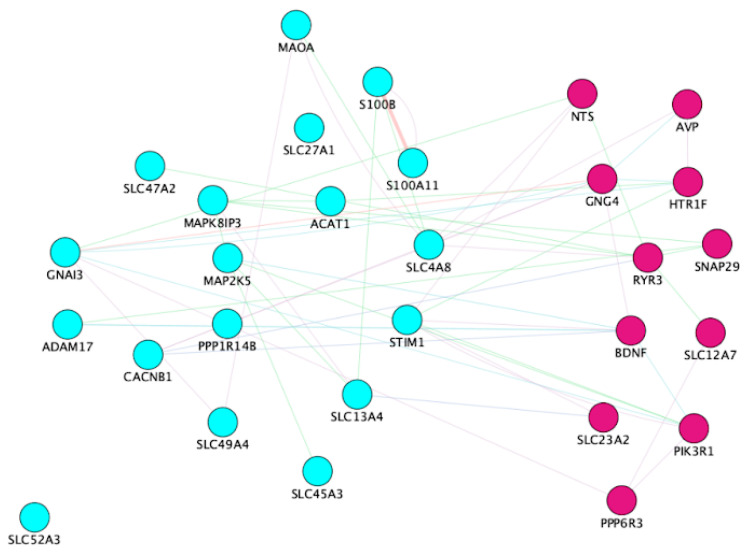
The PPi network of the genes involved in neuropeptide mediated-GPCR signaling (belongs to MEturquoise). These genes play a significant role in the regulation of circadian rhythms, calcium signaling, MAPK signaling. The nodes colored in maroon are upregulated and sky blue are downregulated in the extended photoperiod.

Our IPA and WGCNA analysis revealed several enriched pathways, including GPCR, dopamine, serotonin, melatonin degradation, myelination, tachykinin signaling, and mitochondrial dysfunction. Using KEGG terms, we identified key molecules in these pathways to understand potential mechanisms of microbiota–host crosstalk. The most enriched genes were associated with calcium signaling, MAPK signaling, circadian entrainment, and chemokine signaling (Figure 7). Clustering analysis (Figure 3) demonstrates that these enhanced functional processes collectively invoke a complex interplay of signaling pathways, with calcium signaling emerging as a central component. Calcium signaling plays a fundamental role in various cellular responses to environmental stimuli, including plant defense systems, highlighting its importance in diverse biological contexts [79]. It also regulates cell cycle progression in response to abiotic stress [80]. Furthermore, reactive oxygen species (ROS) in mitochondria trigger monoamine-induced calcium signals, influencing physiological and pathophysiological responses to dopamine [81]. Calcium signaling is versatile in numerous cellular functions and crucial for translocating nuclear proteins like PKC-γ [82]. The calcium signaling is a critically important phenomenon that regulates synaptic activity in the nervous system. Additionally, synaptic activity is the key feature of circadian oscillations, and changes in circadian rhythms alter synapse numbers. Calcium is the most important signaling molecule that plays a role in nervous excitability and regulation of the biological clock [83]. Calcium signaling is intricately linked to circadian rhythms, playing a significant role in regulating the molecular clock and coordinating various physiological processes throughout the day. Circadian rhythms in the SCN neurons are synchronized with calcium rhythms, indicating a causal relationship between intracellular calcium dynamics and the generation of circadian rhythms [84]. Furthermore, regulating voltage-dependent calcium channels (VDCCs) by circadian mechanisms is crucial for maintaining rhythmic clock gene expression in the SCN, highlighting the importance of calcium signaling in the molecular machinery of circadian clocks [85]. Additionally, mitochondrial calcium signaling has been implicated in mediating rhythmic extracellular ATP accumulation in SCN astrocytes, further emphasizing the role of calcium in coordinating circadian processes at the cellular level [86]. These enriched pathways point towards key homeostatic processes. These pathways play a role in intracellular calcium signaling, which is crucial for activating lymphocytes and neurological functions (KEGG hsa04020). The gene regulatory network of differentially expressed genes involved in these pathways is shown in Figure 8. The genes in the maroon-colored PPI network in Figure 8 were found to be upregulated and blue-colored were downregulated in extended photoperiods.

The interaction between circadian rhythms and the gut microbiome may include calcium signaling as a key mediator. Calcium signaling regulates neurotransmitter release and cellular functions essential for circadian rhythm generation [87]. The influx of calcium ions into the presynaptic terminal triggers the release of neurotransmitters into the synaptic cleft, facilitating signal transmission between neurons [88]. This process is tightly regulated and involves various proteins and signaling pathways. For example, synaptotagmins, calcium sensors on synaptic vesicles, are vital for vesicle fusion with the presynaptic membrane and subsequent neurotransmitter release [89]. Calcium signaling also modulates synaptic plasticity, where changes in calcium levels can impact the strength of synaptic connections and neuronal communication [90]. On the other hand, the gut microbiome can be influenced by factors such as diet, disease, and probiotics, impacting cognitive function and overall health [91]. Given the bidirectional communication between the gut microbiome and the host, calcium signaling may serve as a signaling pathway through which circadian rhythms interact with the gut microbiome. Furthermore, the gut microbiome’s role in modulating host metabolism and immune function suggests a potential link between calcium signaling, circadian rhythms, and gut microbiome-mediated effects on health and disease.

## 4. Conclusions

This study provides novel insights into how photoperiods and circadian rhythms modulate gene expression in the chicken brain. A key finding is the substantial alteration of neurochemical and mitochondrial pathways under extended photoperiod conditions, evidenced by the downregulation of pathways related to dopamine degradation, mitochondrial dysfunction, and G-protein coupled receptor signaling. This highlights a previously underappreciated link between extended light exposure and critical neurophysiological processes necessary for maintaining cellular homeostasis.

Through DiscoRhythm analysis, we identified 723 genes with pronounced circadian oscillations, underscoring a robust and pervasive circadian regulation of metabolic and intracellular processes. Notably, differential regulation of key circadian clock genes and GPCR receptors reveals a novel interaction where extended photoperiods profoundly affect neurotransmitter and cytokine signaling pathways. The identified associations between circadian rhythms and pathways such as Aldosterone-regulated sodium reabsorption, GABAergic synapse function, and oxidative phosphorylation further substantiate the innovative perspective on dopamine’s dual regulatory role and mitochondrial function in circadian biology.

Moreover, our integrative co-expression analysis offers groundbreaking evidence of the intricate interplay between the host’s circadian transcriptome and gut microbiota composition. The discovery of distinct gene expression modules (MEbrown, MEturquoise, MEblue, and MEgrey) associated specifically with microbial taxa advances our understanding of microbiota–host interactions. This points to a previously unrecognized potential for microbiota-driven modulation of circadian-regulated pathways, including cholesterol biosynthesis, mitochondrial integrity, dopamine signaling, and melatonin metabolism.

Collectively, this work establishes a foundation for future functional studies into the mechanisms linking circadian biology, microbiota composition, and host physiological functions in avian models. It highlights promising strategies for targeted microbiome interventions aimed at optimizing circadian health, with significant implications for metabolic and immune system homeostasis.

## Figures and Tables

**Table 1:** Provides a quality control summary of RNASeq Reads obtained from samples of both normal photoperiod (NP) and extended photoperiods (EP).

QC	NP_ZT0	NP_ZT24	NP_ZT48	EP_ZT0	EP_ZT24	EP_ZT48
Total M Seqs	21.48	16.43	15.31	16.25	14.66	17.84
Length	73	73	73	73	73	73
M Aligned	18.81	14.8	13.24	14.32	12.78	14.55
% Aligned	87%	90%	86%	88%	87%	84%
M Assigned	10.54	8.29	6.93	6.96	6.34	6.99
% Assigned	47%	50%	44%	43%	42%	41%

## Data Availability

The microbiota data is available in a publicly accessible repository at: DOI 10.6084/m9.figshare.6938249.v1. The RNAseq data is available on request from the authors.

## Supplementary Materials

The following supporting information can be downloaded at: https://www.mdpi.com/article/10.3390/microorganisms13040789/s1, Table S1: Output of Cosinor algorithm in Discorythm; Table S2: Output of JTK-cycle algorithm in Discorythm; Table S3: Module–microbiome relationship calculated by WGCNA.
